# Supplementary material for: Accurate predictions of SARS-CoV-2 infectivity from comprehensive analysis
Source: eLife. 2024 Dec 24;13:RP99833. doi: 10.7554/eLife.99833 (PMC11668528; doi:10.7554/eLife.99833)
Supplement: Supplementary file 2. [file elife-99833-supp2.pdf]

Sign up & Login

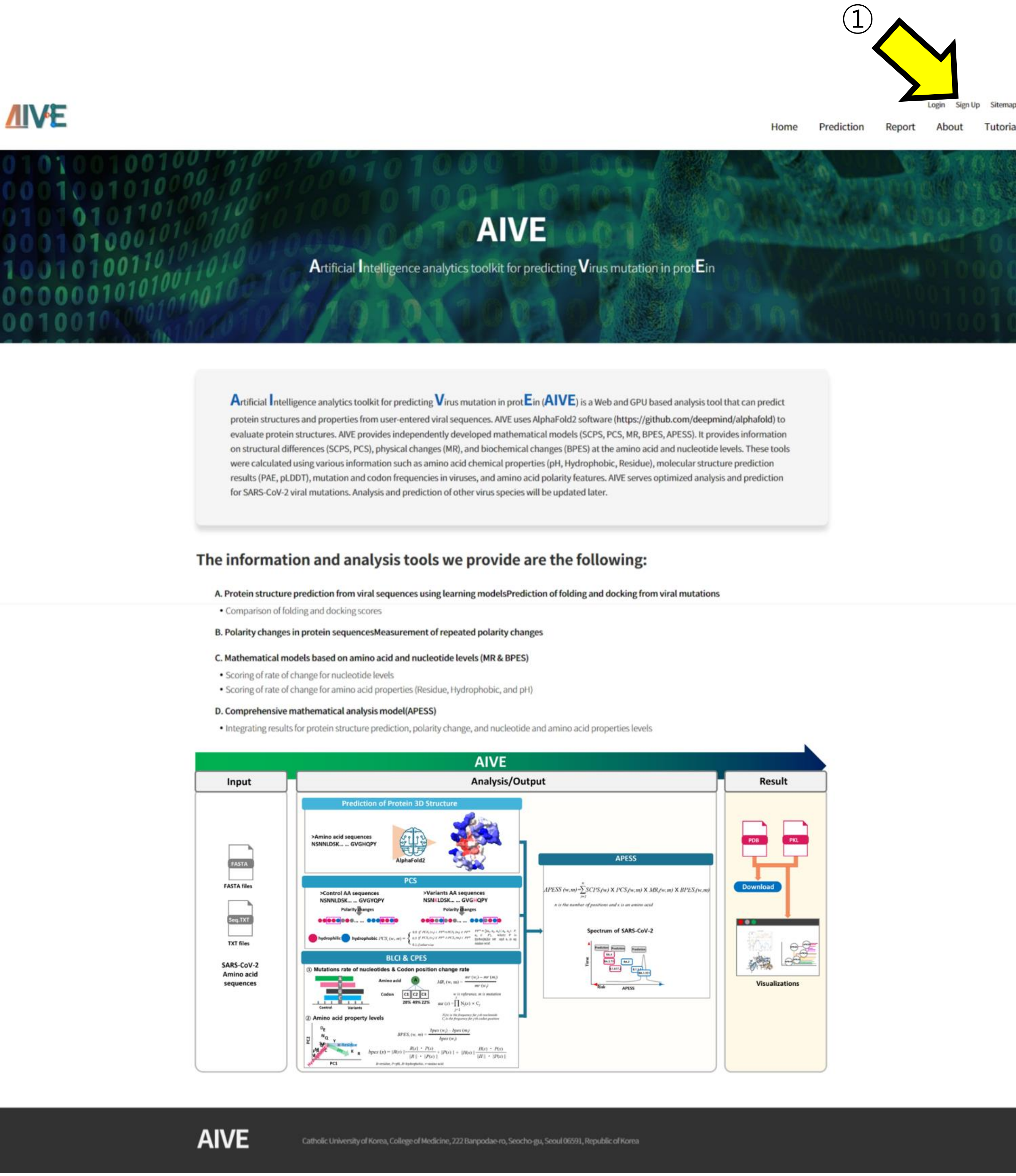

AIVE is used by creating a personal account for the ease of comparing analysis results.
① Click on the "Sign Up" option to proceed with the account creation.

Sign up & Login

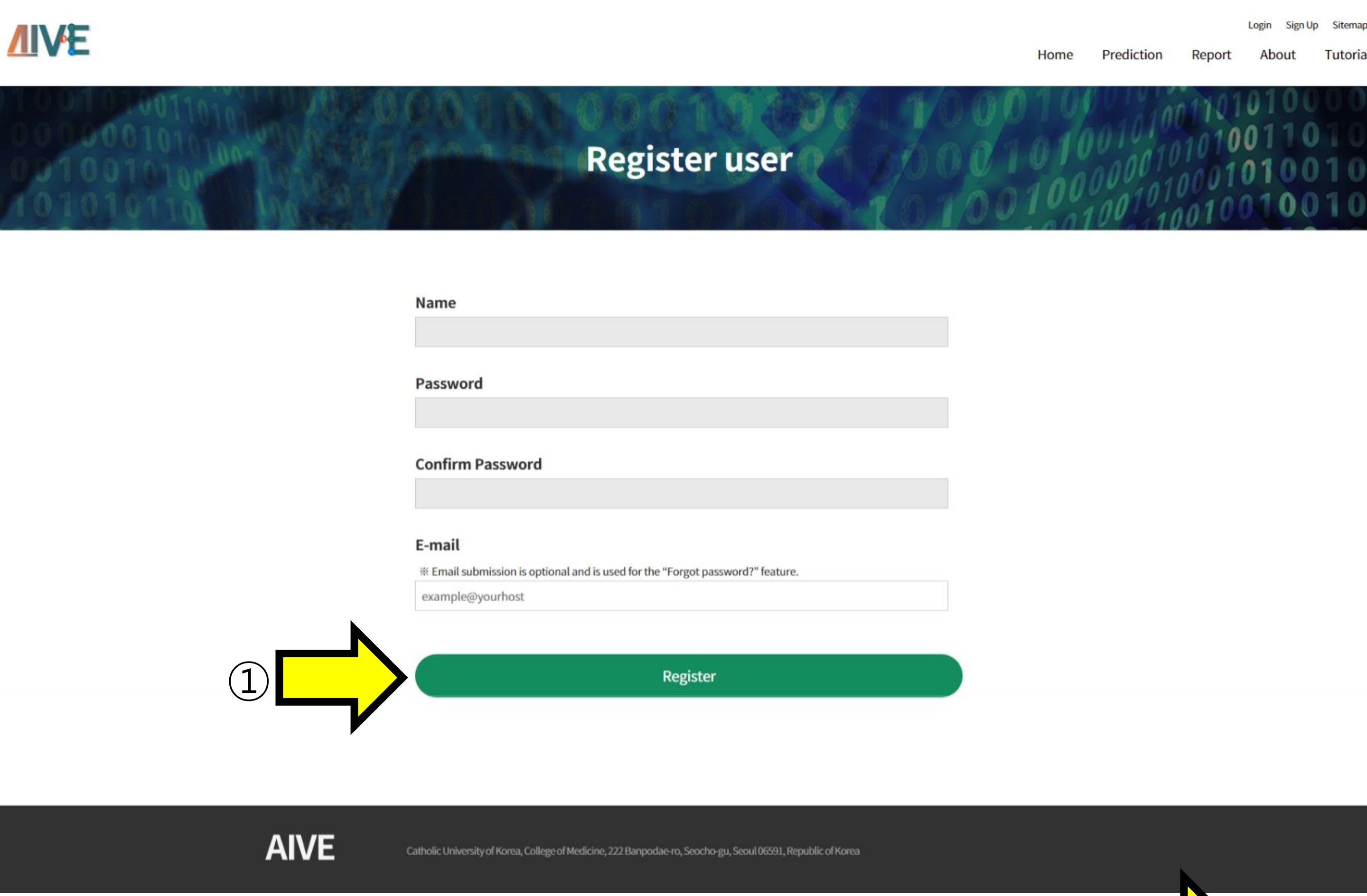

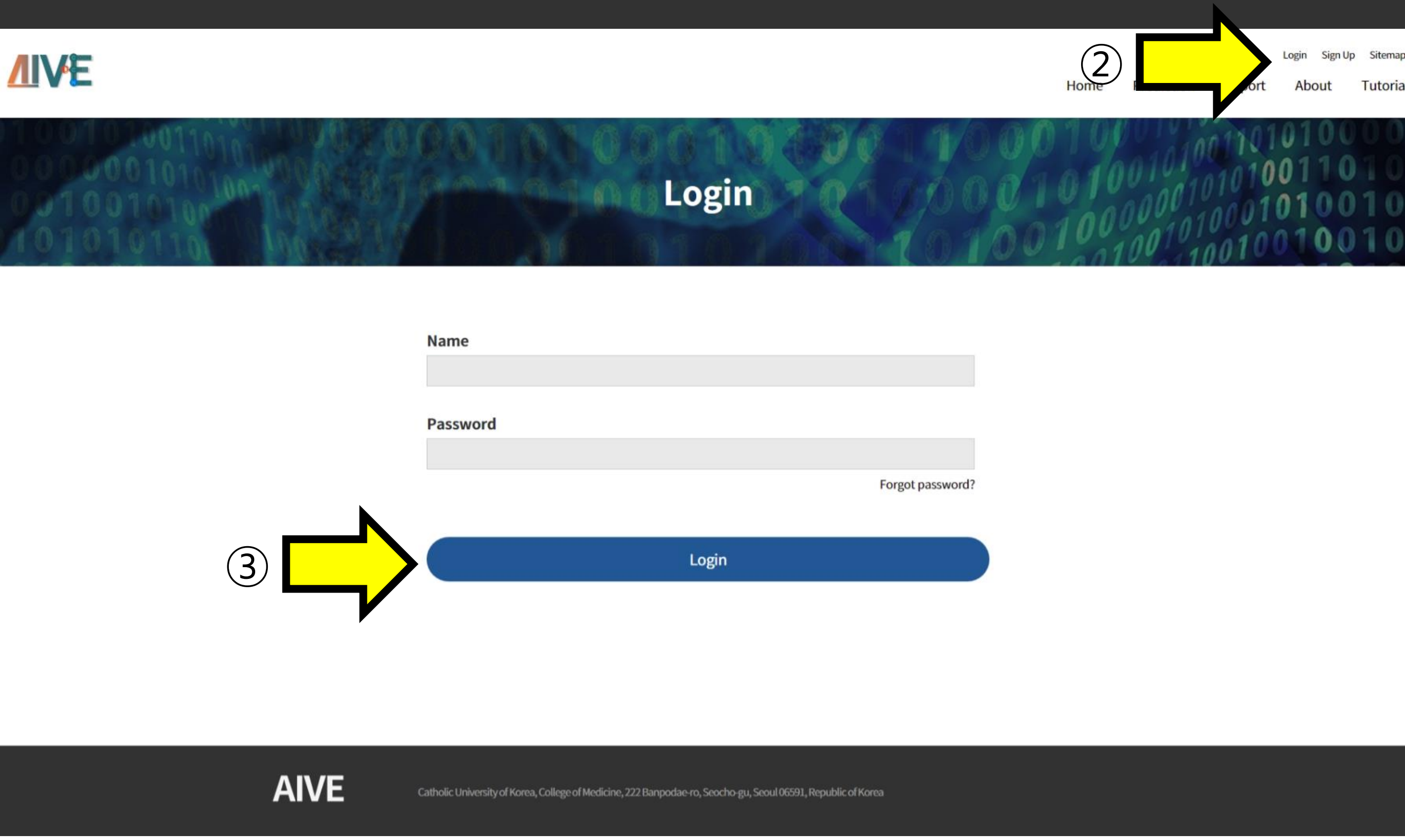

① Enter the username and password. To use the "Forgot password?" feature, enter your email and proceed with account creation.
②, ③ Access the "Login" section, enter the account information, and log in.

Demo

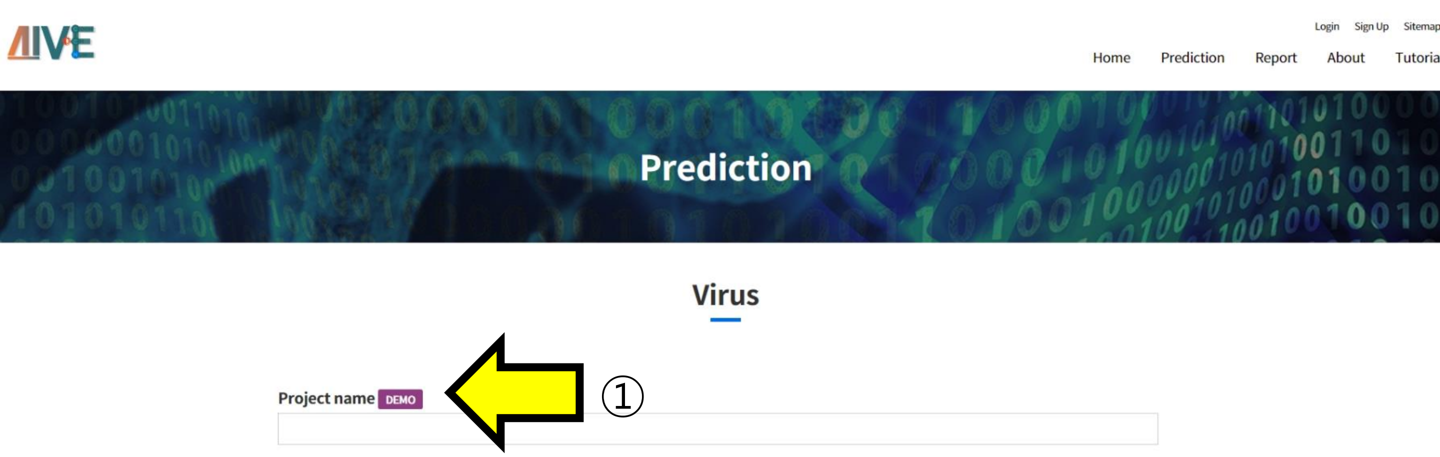

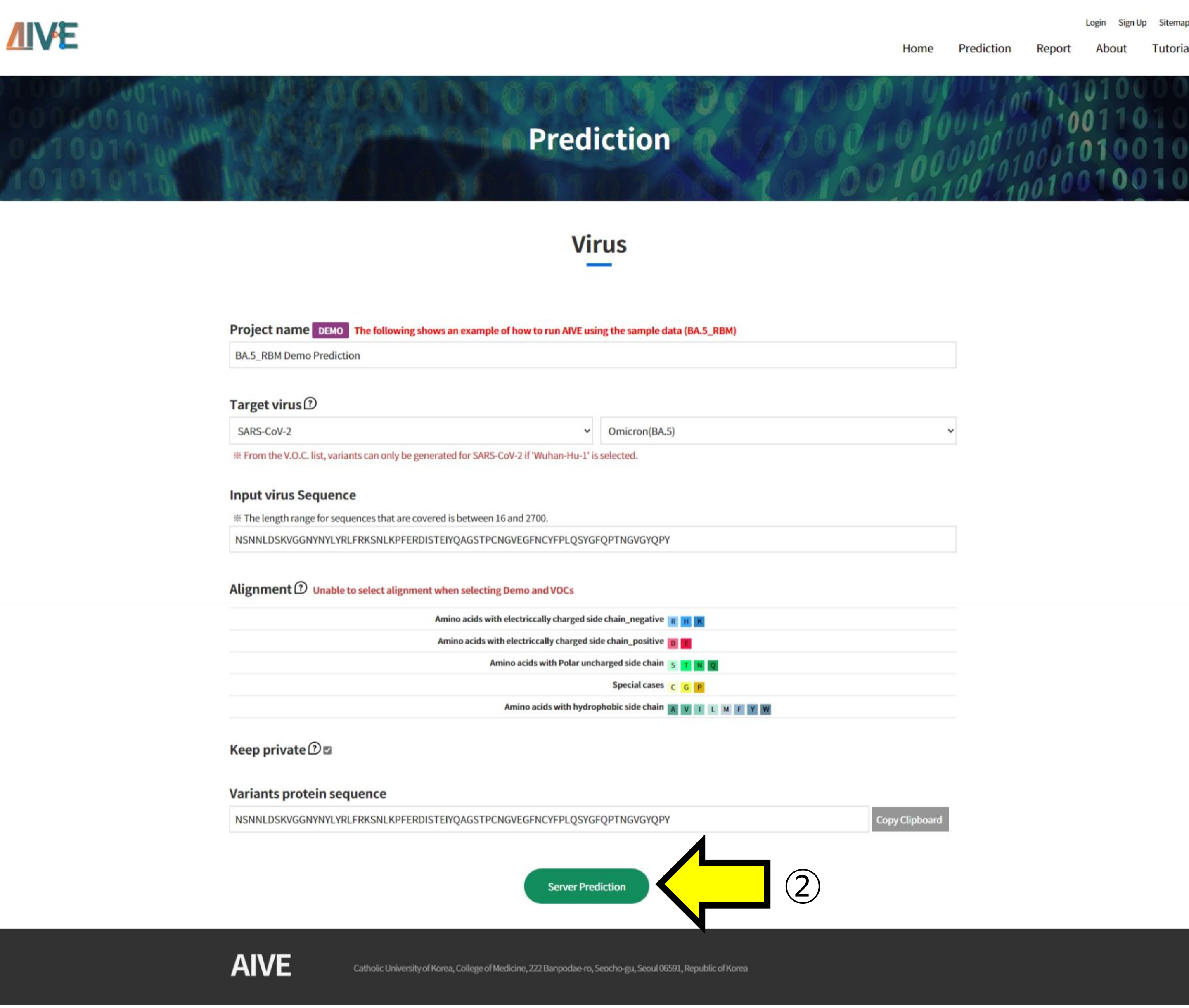

AIVE provides a demo feature that demonstrates the analysis of SARS-CoV-2 variants of concern (VOCs).
① Clicking on the Demo button will automatically select the RBM [S:437-508] region of the SARS-CoV-2 BA.5 variant.
② To predict and analyze the structure of the sequence generated by the Demo function, click the "Server Prediction" button to submit the task.

Predict VOC structure

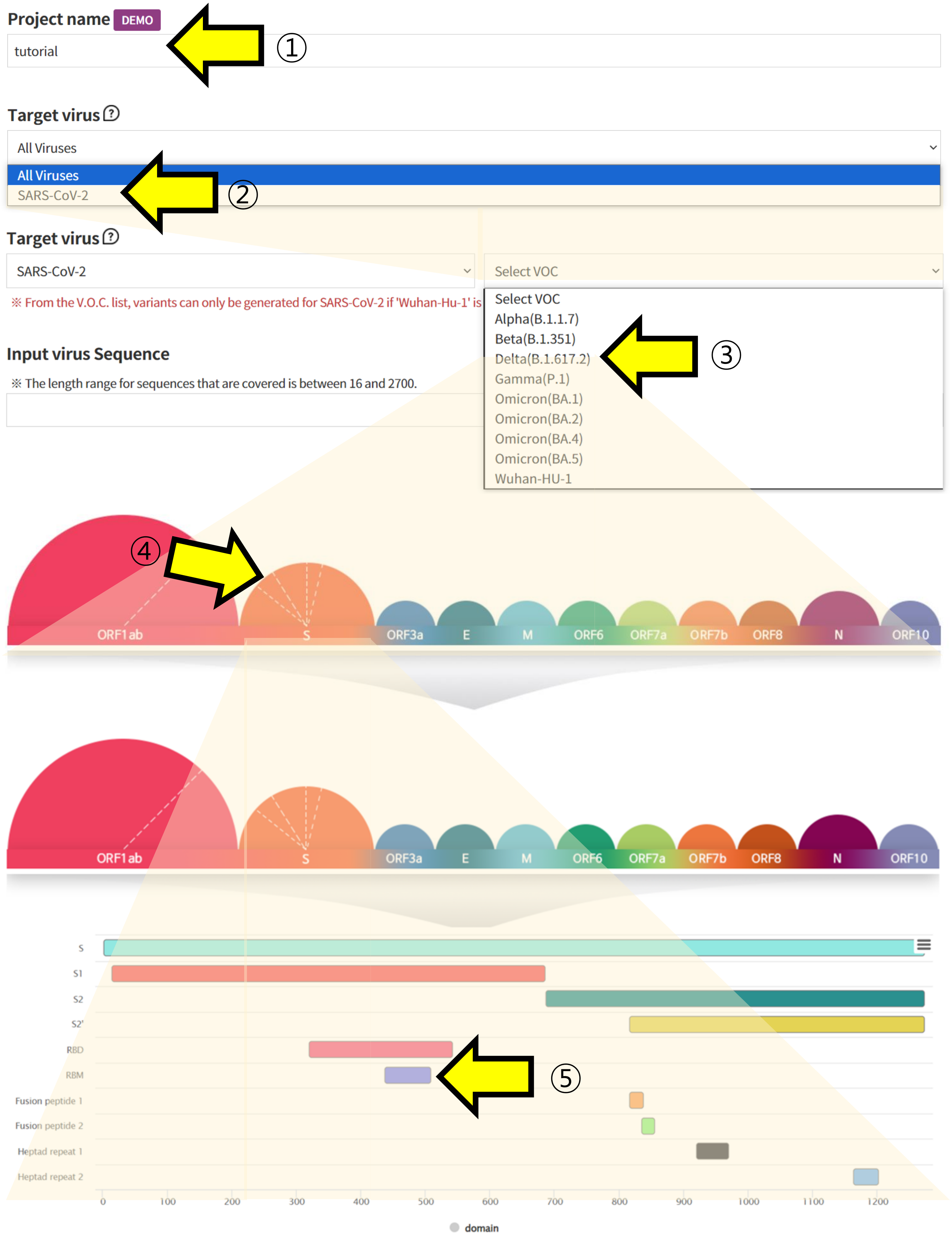

In AIVE, users can personally select and analyze the VOC and domains of SARS-CoV-2.
① Write the Project name for task categorization.
② Select SARS-CoV-2 in the Target Virus section to retrieve information on Coronaviruses.
③, ④, ⑤ Choose the VOC and regions you want to analyze to retrieve the corresponding Amino Acid sequence information.

Submit

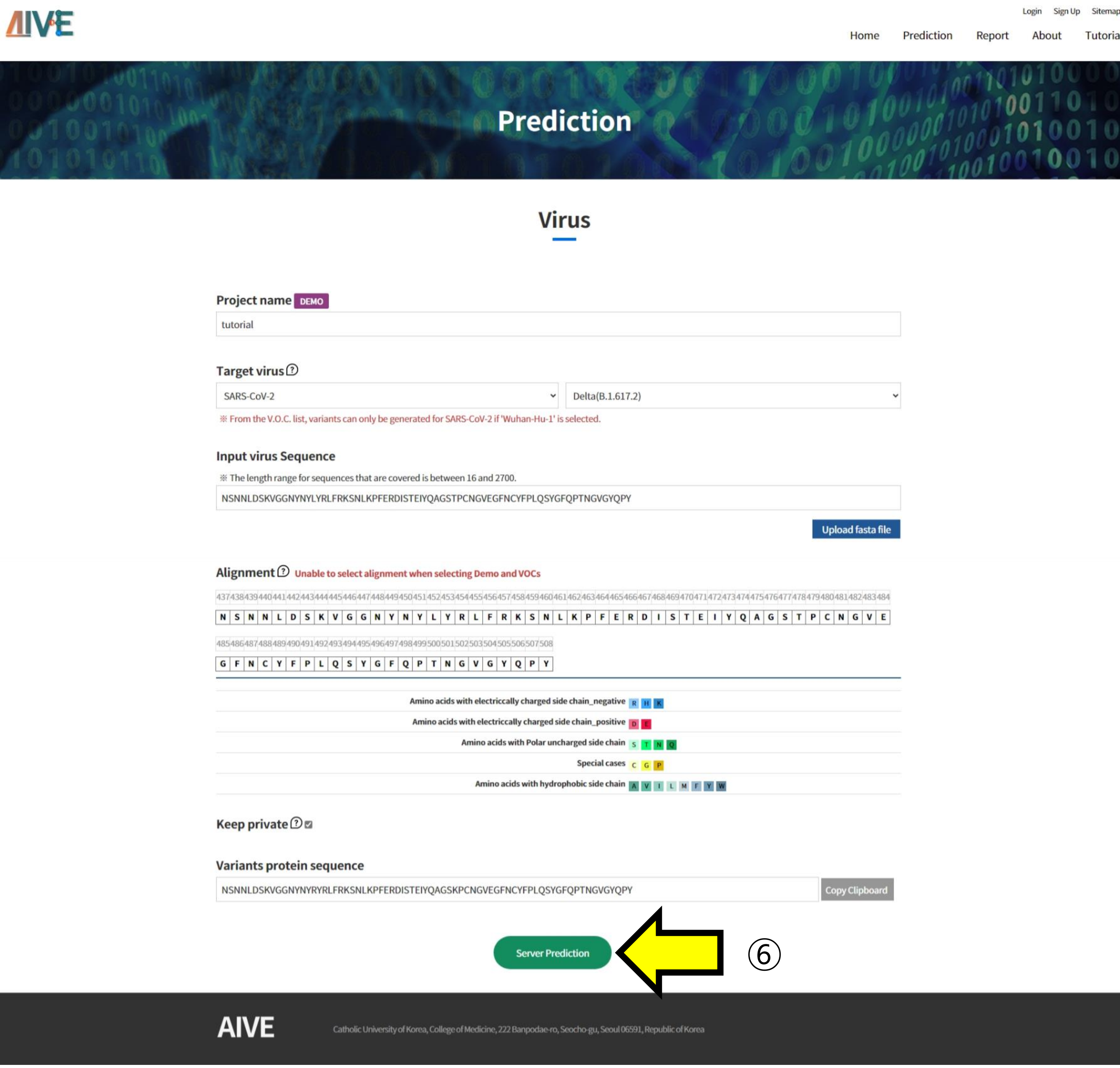

⑥ Click the "Server Prediction" button to start the prediction and analysis of the Project

List

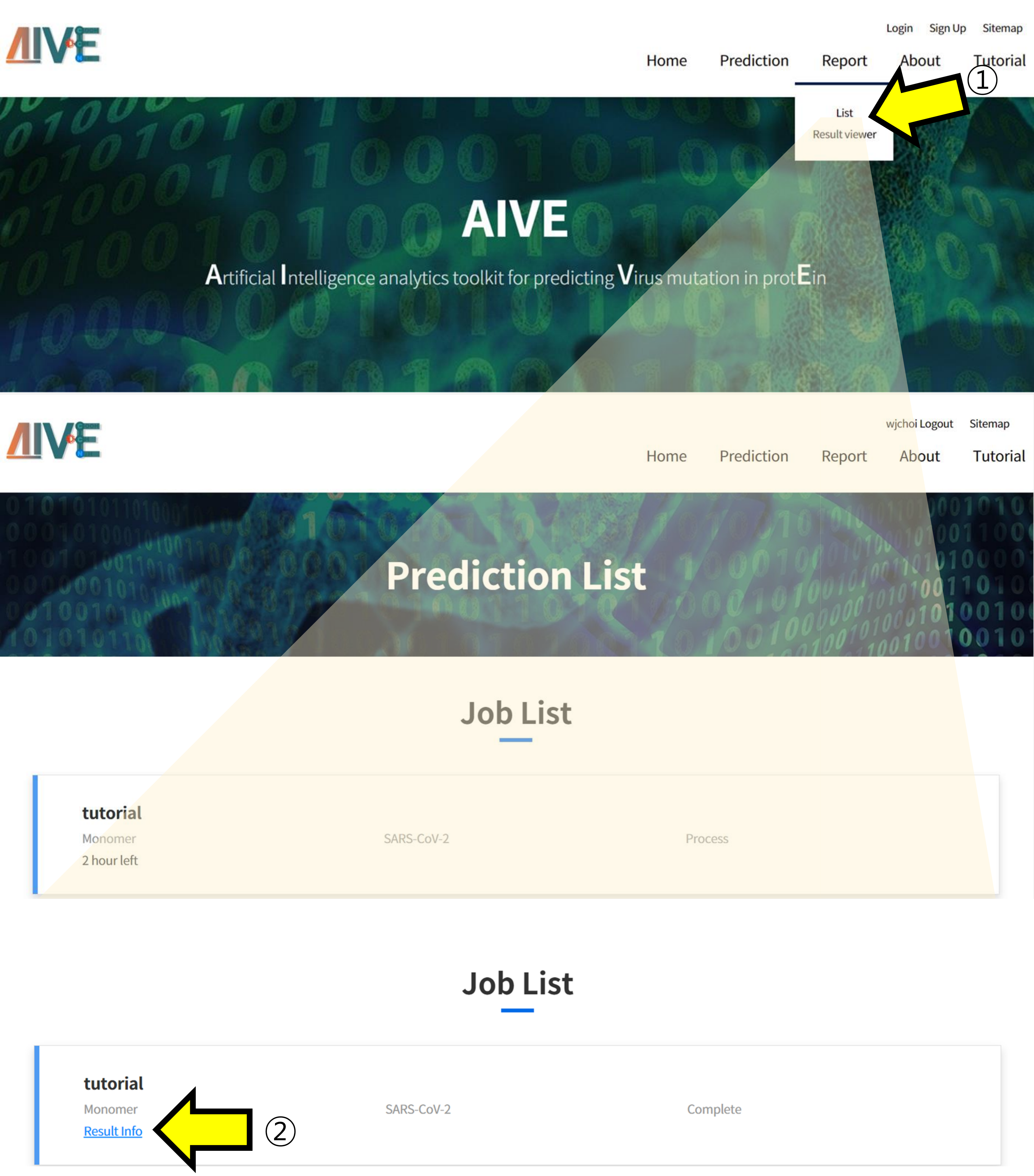

In the "List" section, users can check the list of submitted Projects and their progress.
① Click the "Report menu" and then select "List" from the submenu to check Projects.
② You can check the analysis results by clicking on the "Result info" section for completed tasks.

Generate SARS-CoV-2 mutated sequence

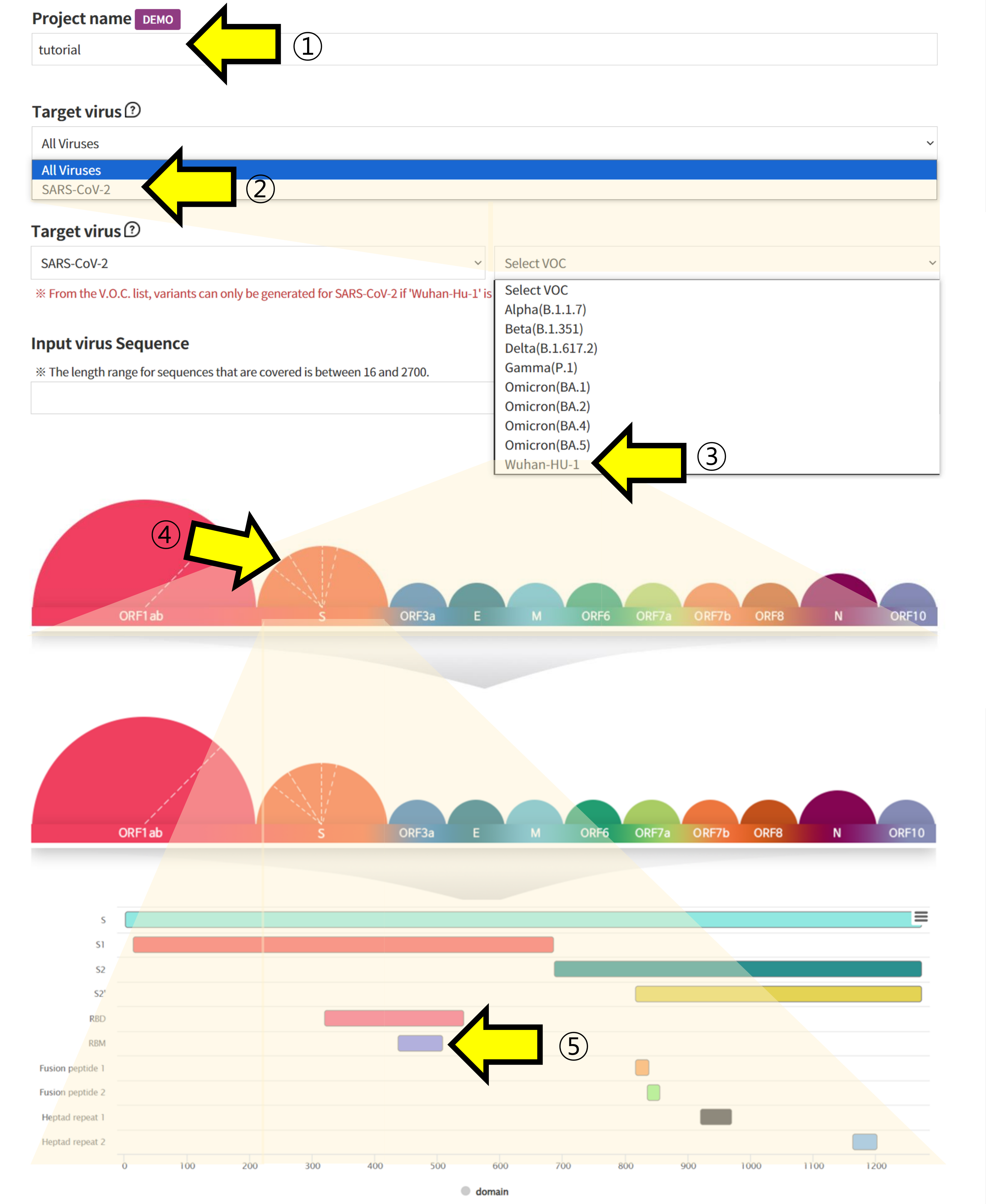

Users can not only access information about VOCs but also generate mutations for analysis.
① Specify a Project name to categorize the submitted task
②, ③, ④, ⑤ Choose Wuhan-HU-1 sequence of SARS-CoV-2 and retrieve the sequence of the region you want to verify.

Generate SARS-CoV-2 mutated sequence

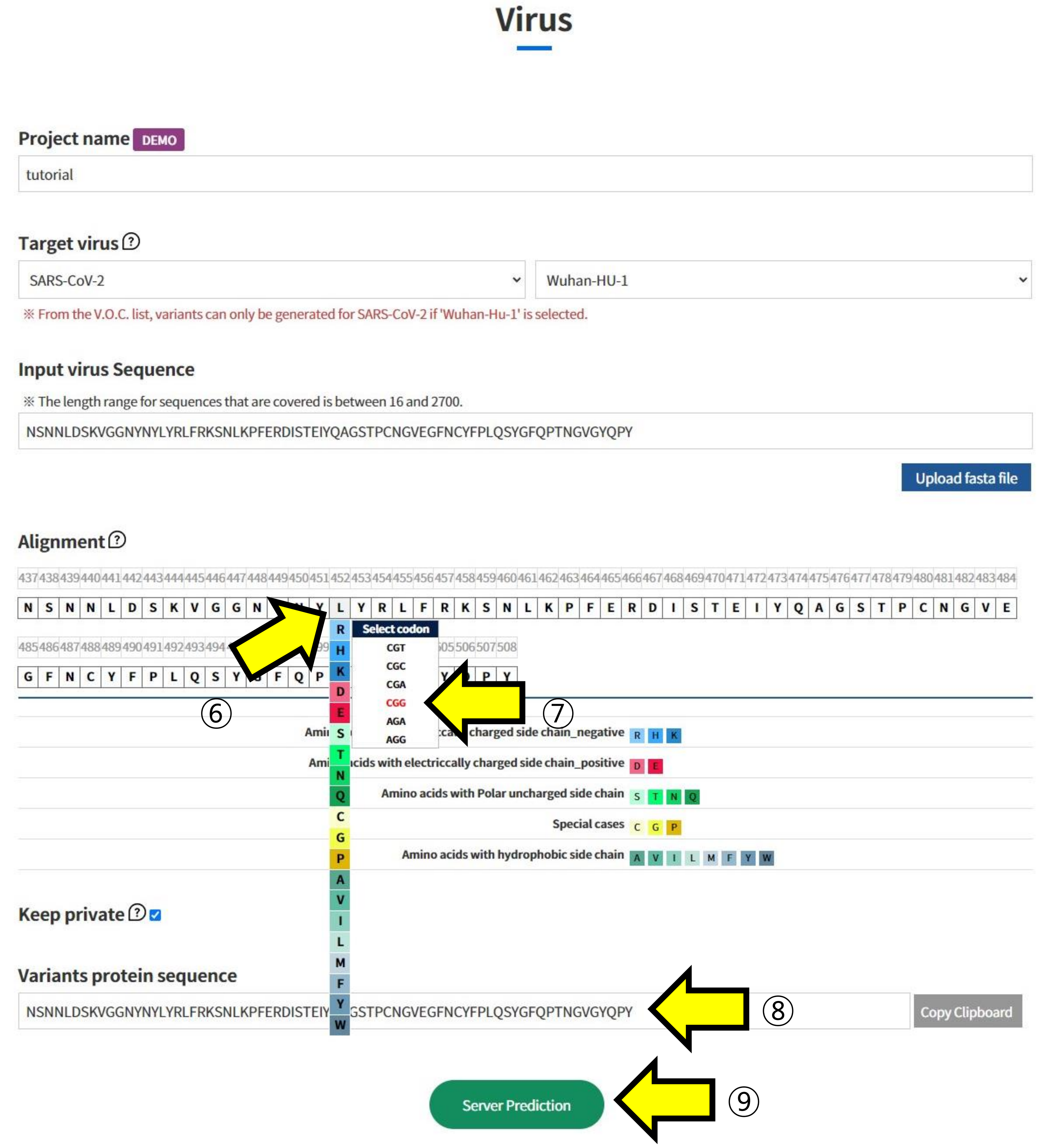

⑥ In Alignment, click on the positions in the sequence of Wuhan-HU-1 to select the mutated Amino Acid.
⑦ Additionally, choose which codon to mutate into from the selected Amino Acid. This function is used to assess the impact of mutations at the codon level.
⑧ The sequence with the selected mutations is displayed.
⑨ Submit the task to proceed with the structural prediction and analysis of the mutated sequence.

User sequence - Monomer

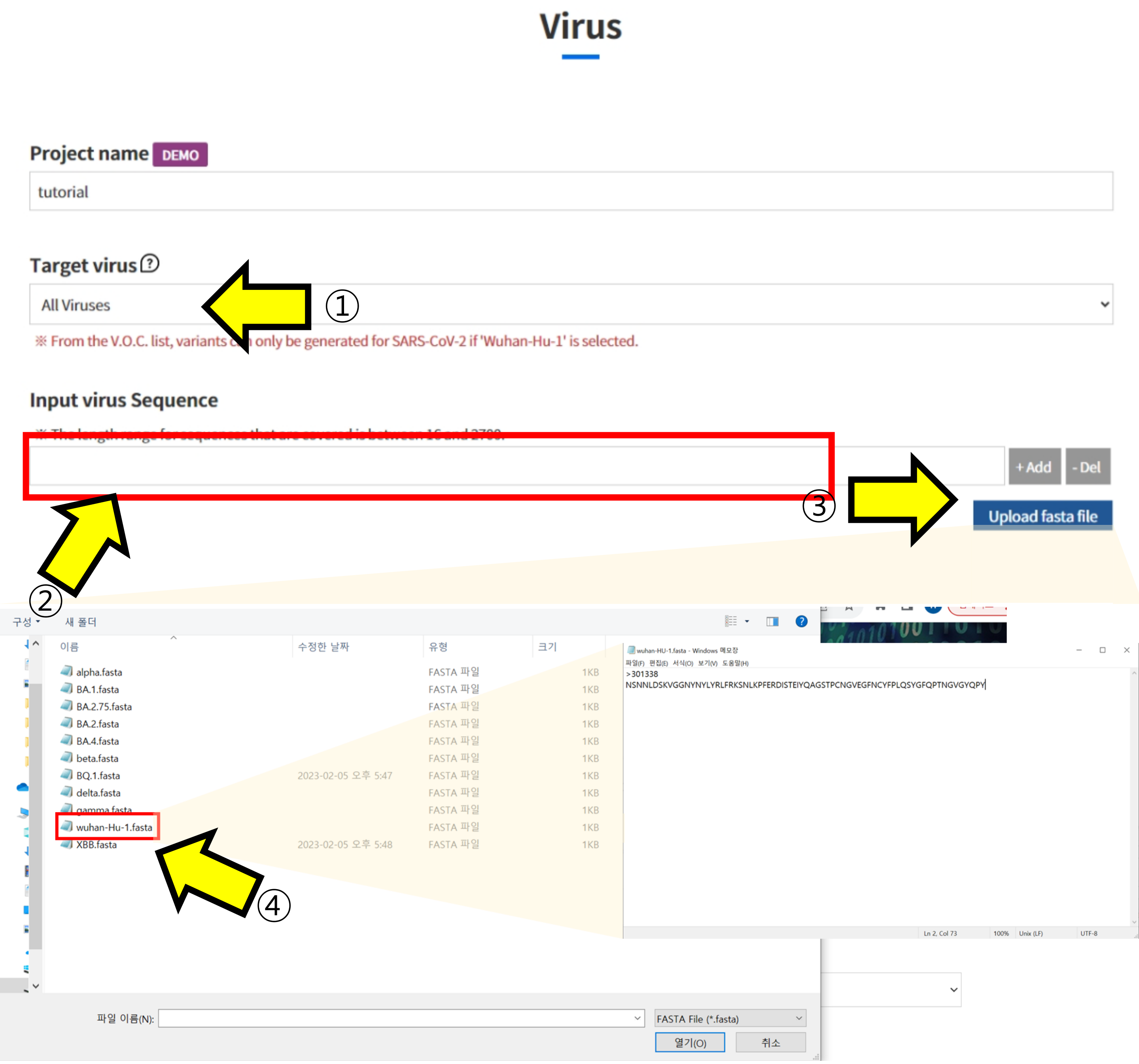

In AIVE, users can predict the structure of sequences, not limited to SARS-CoV-2. Let's take a look at monomer structure prediction

① Select "All viruses" in the Target Virus section.
② Enter the Amino Acid sequence you want to check in the "Input virus Sequence" box.
③ Alternatively, you can upload a fasta file instead of entering it directly.
④ When the upload window appears, select the fasta file you want to check to pull up the sequence.

User sequence

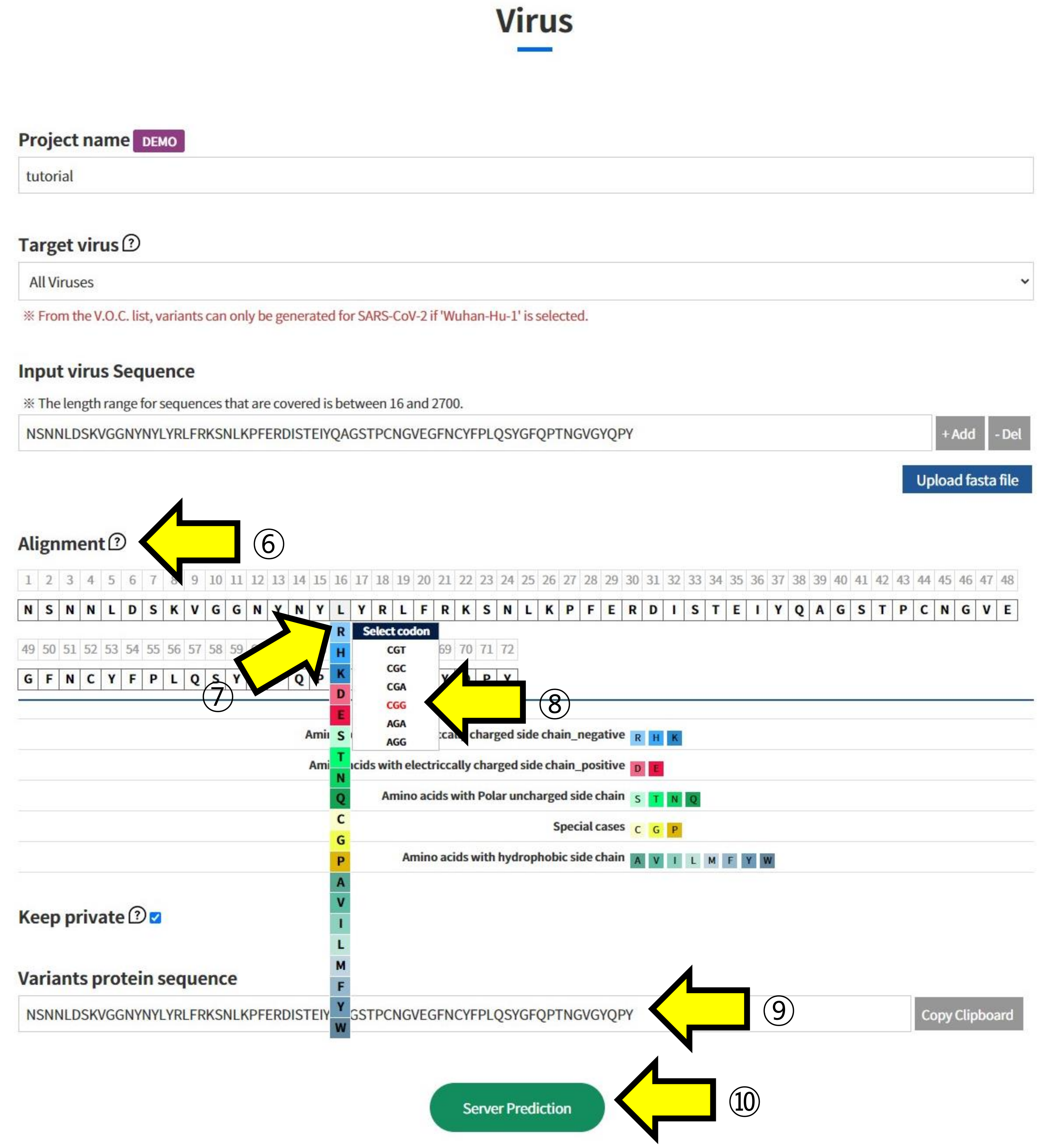

⑥ The Amino Acid sequence recorded in the fasta file you uploaded is displayed in “Alignment”.
⑦ Click on the positions in the displayed sequence where you want to introduce mutations and select the mutated Amino Acid.
⑧ Additionally, choose which codon to mutate into from the selected Amino Acid. This function is used to analyze the impact of mutations at the codon level.
⑨ The sequence with the selected mutations is displayed.
⑩ Submit the task to proceed with the structural prediction and analysis of the mutated sequence.

User sequence - Multimer

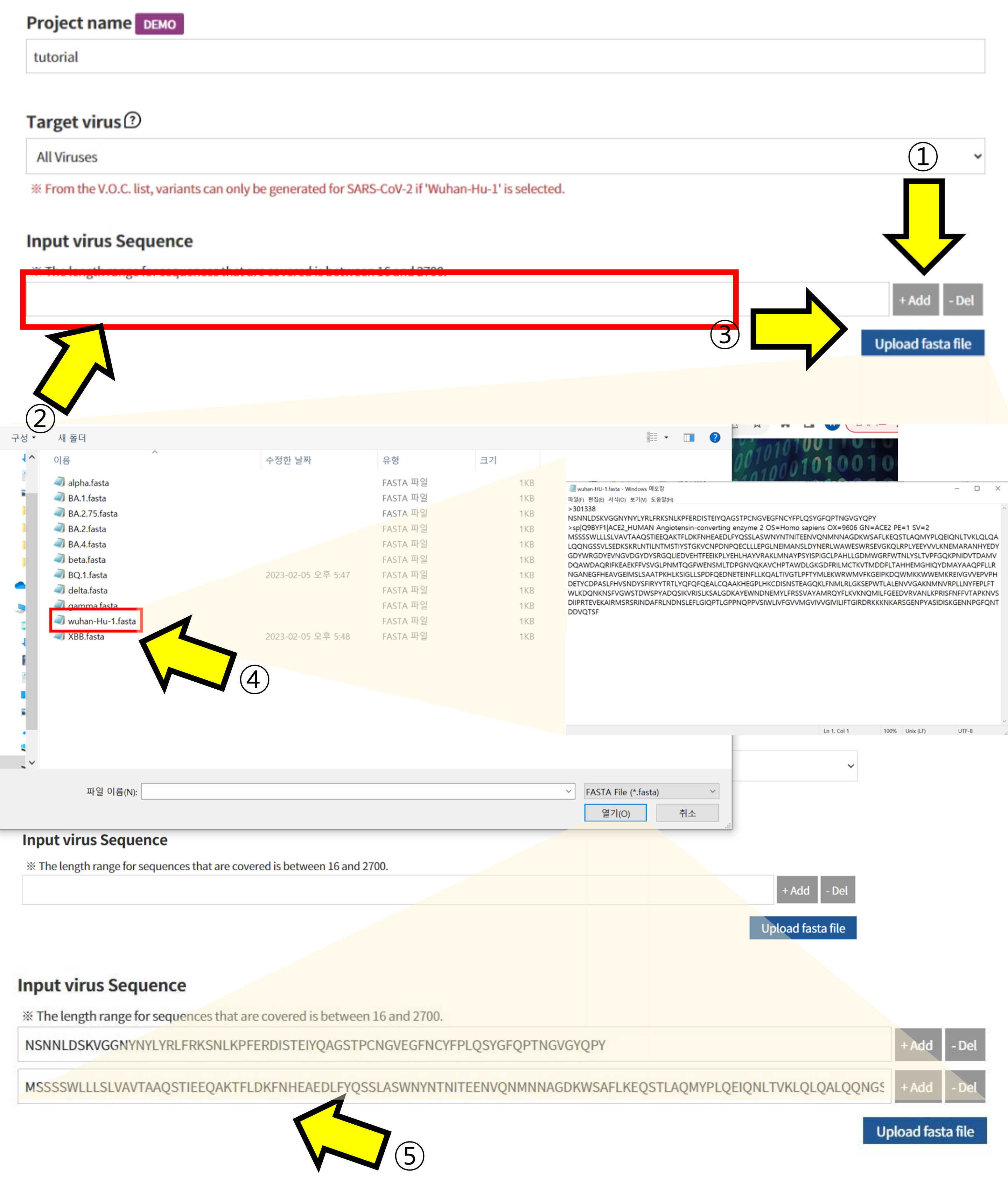

Let's look at the case of predicting a Protein Complex structure:
① Click the +Add button to create as many sequence input boxes as there are chains in the protein complex you want to predict.
② Enter the Amino Acid sequence of each chain in the generated “Input virus Sequence” boxes.
③ Alternatively, a fasta file can be uploaded without entering the sequence directly
④ When the upload window appears, select the fasta file to retrieve the sequence.
⑤ The sequences of each chain, as stored in the fasta file, are inputted.

User sequence - Multimer

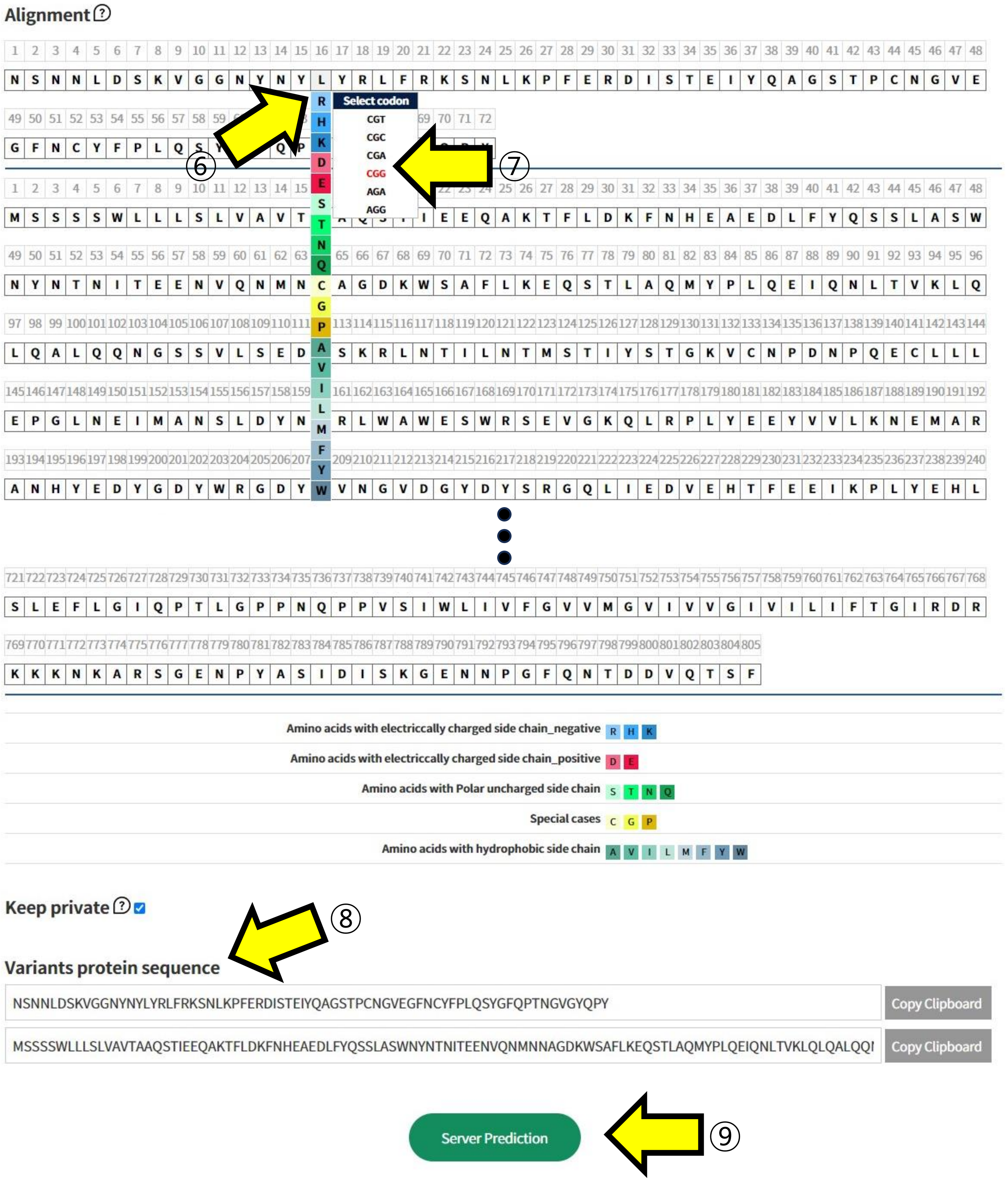

⑥ Click on the positions in the displayed sequences where you want to introduce mutations and select the mutated Amino Acid.
⑦ Additionally, choose which codon to mutate into from the selected Amino Acid. This function is used to analyze the impact of mutations at the codon level.
⑧ The sequence with the selected mutations is displayed.
⑨ Submit the task to proceed with the structural prediction and analysis of the mutated sequence.

Result report page - Structure

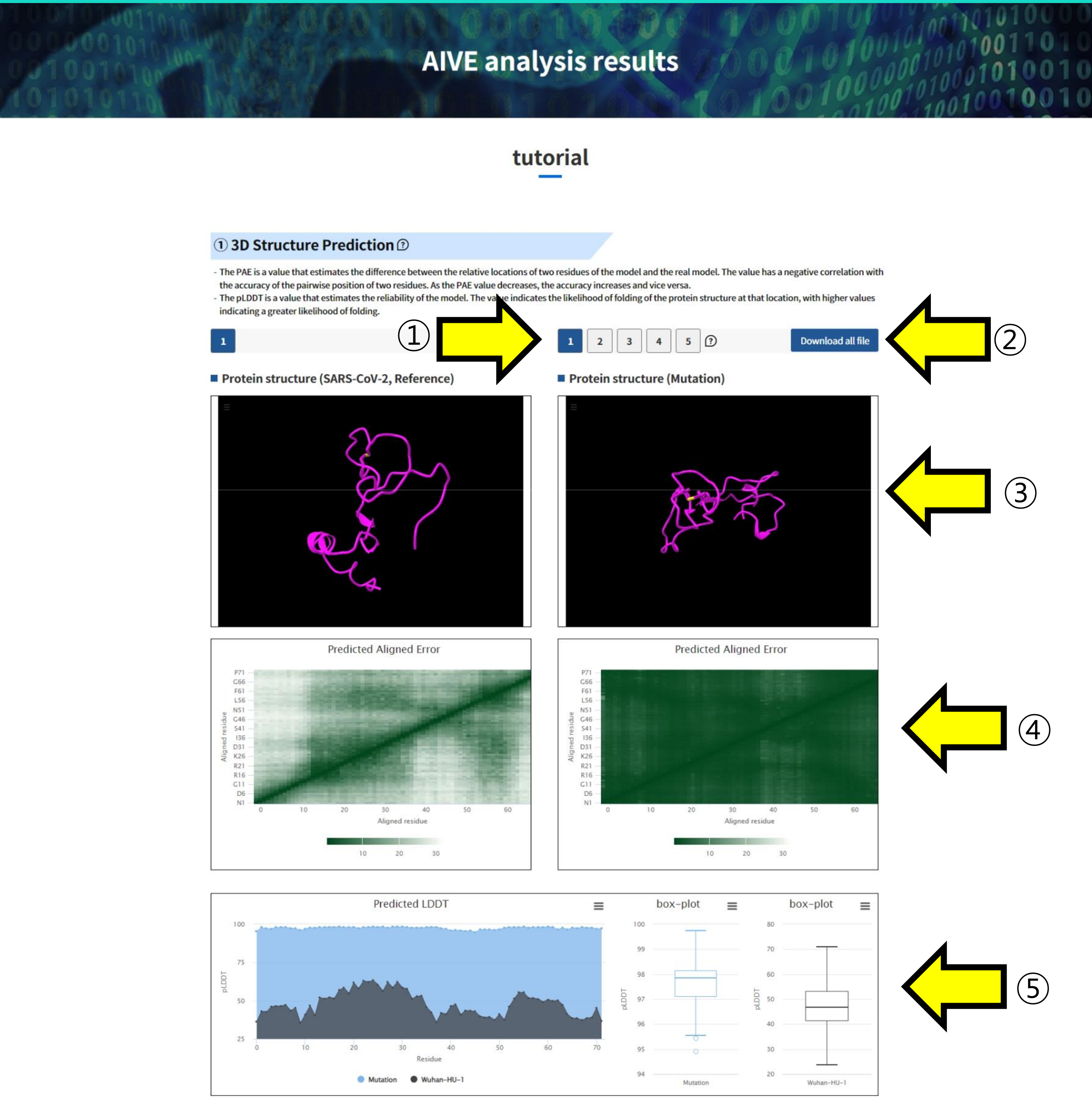

From the prediction and analysis results of SARS-CoV-2, the 3D structure prediction results can be accessed.
① You can select and view the predicted 3D structures by choosing from the five available options.
② Use the "Download all file" button to download the result files of the predicted structures to your device.
③ You can visualize the predicted 3D structure for inspection and comparison with SARS-CoV-2 Wuhan-HU-1. ④, ⑤ Clicking on the highlighted regions in positions allows you to inspect them.
④ Predicted aligned error (PAE) is a value that estimates the difference between the relative locations of two residues of the model and the real model. A low PAE value indicates that the accuracy of the relative location of the two residues is high.
• The color at (x, y) indicates AlphaFold's expected position error at residue x if the predicted and true structures were aligned on residue y.
• If the PAE is generally low for residue pairs x, y from two different domains, it indicates that AlphaFold predicts well-defined relative positions and orientations for them. (Explanation from AlphaFold FAQ)
⑤ Predicted LDDT(pLDDT) is a value that estimates the reliability of the model. It estimates how well the actual model residue and predicted model residue match. At the same time, it indicates how well the protein structure folds in the corresponding location. A low pLDDT value indicates that the reliability of the corresponding position is low and that it possesses a disordered structure.

Result report page - APESS

② Structure difference graph according to position

- Protein structure prediction characteristics (SCPS: SubClustering of Protein Structure in ①) and polarity (PCS: Polarity Change Score) are shown.
- SCPS divides the residues constituting the 3D protein structure into several groups using K-means clustering. The indicated groups are those with a high proportion of WHO name variants.
- PCS assigns weight to a position having a specific structure (where P appears consecutively) by polarity features of amino acid.
- Ratio change in frequencies of amino acids sequences (mr: mutation rate) and Biochemical properties of amino acid sequencies (bpes: biochemical properties eigen score) are shown.
- MR is the rate of change of Amino Acid and its constituent nucleotides; the higher the rate, the higher the rate of change.
- BPES measures changes in the biochemical properties of amino acids at the site of a mutation. A high rate of change results in a high BPES value. Biochemical properties are measured by integrating amino acid residue, pH, and hydrophobic information.

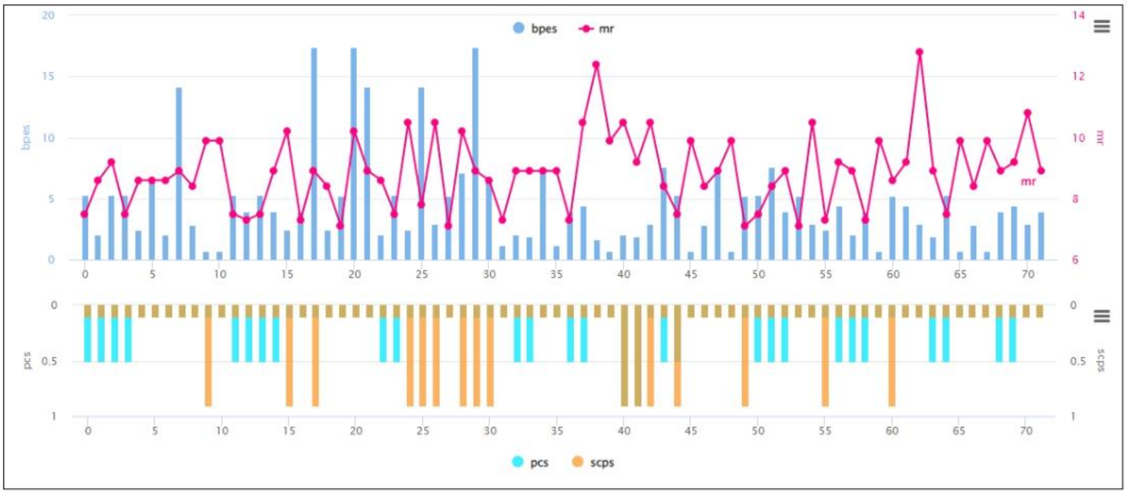

③ APESS according to position

- APESS is an amino acid property eigen selection score calculated by multiplying the values of SCPS, PCS, MR, and BPES.
- APESS is a value that integrates the results of ② and ③. The higher the APESS value, the more dangerous the mutation is.
- The graph shows the positions of mutations with structural differences (SCPS, PCS) in viral proteins. It also shows the positions and magnitudes of relatively large physical changes (MR) and biochemical changes (BPES).

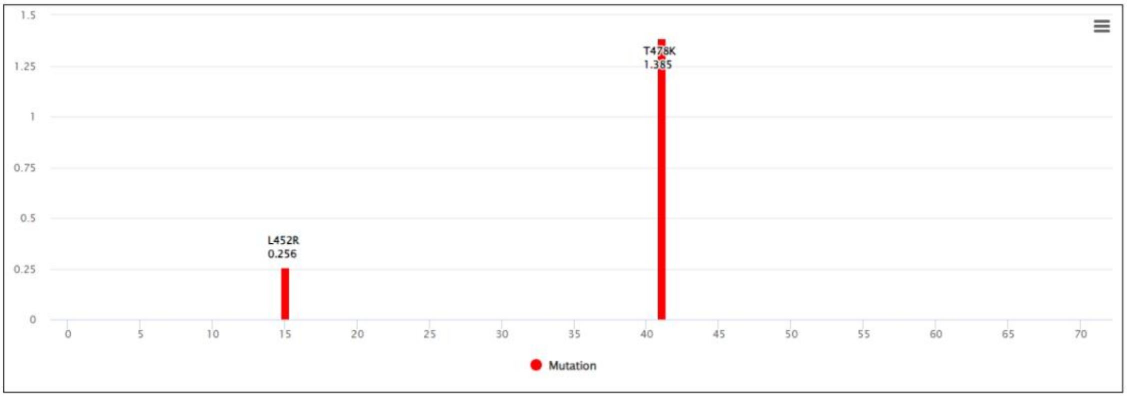

④ APESS distribution graph

- The following graph shows the APESS distribution of mutations generated through random sampling. The red area belongs to the quantile set of 0.05, signifying that the mutation is risky if it belongs to this area. A lettered balloon indicates the score position of the WHO VOC variant, and the red flag indicates the APESS position of the protein predicted in ①.

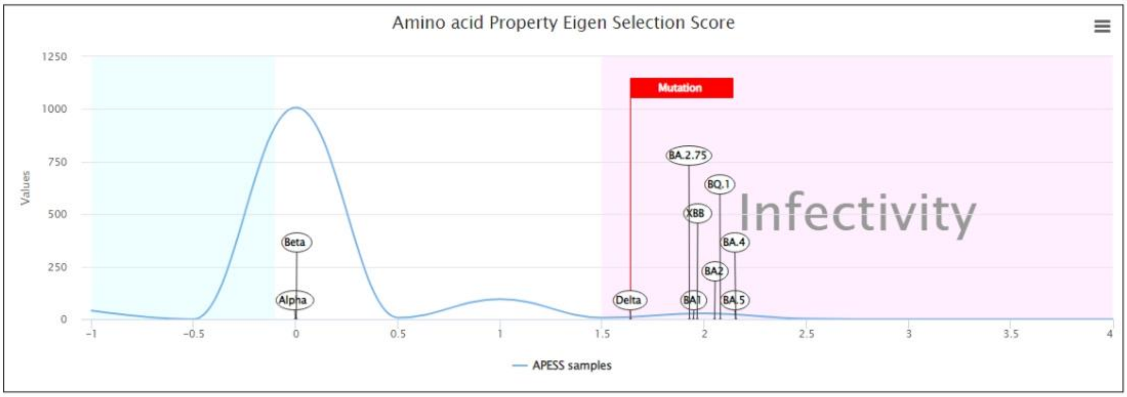

Result report page – APESS subscore

① SCPS

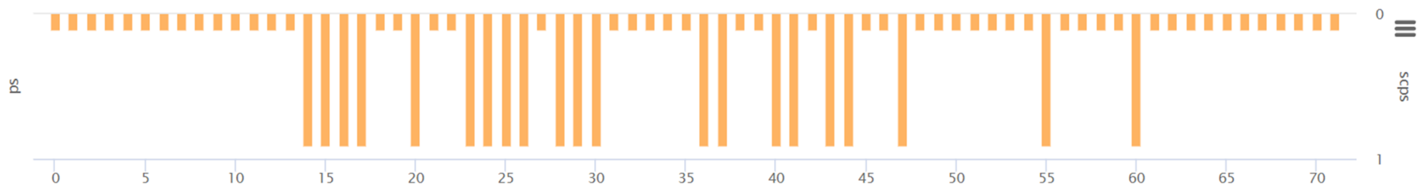

② PCS

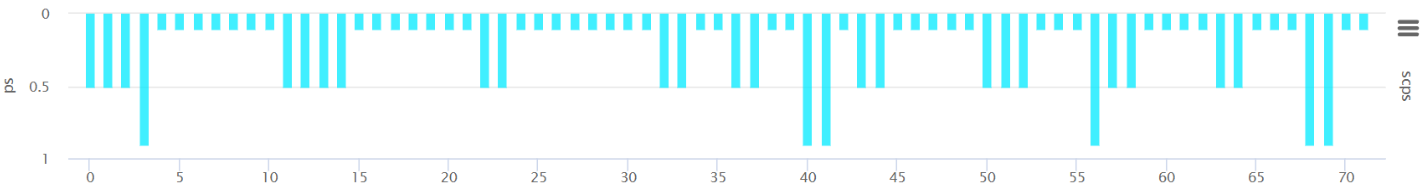

③ BPES

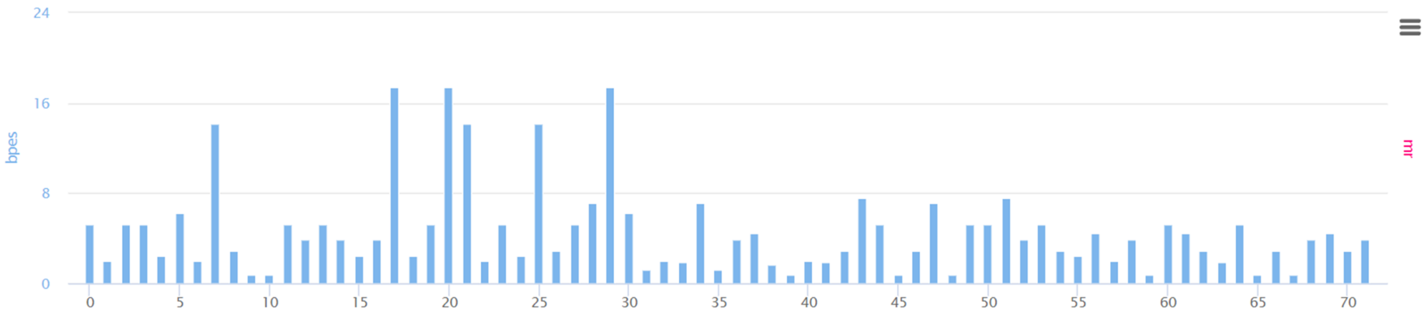

④ MR

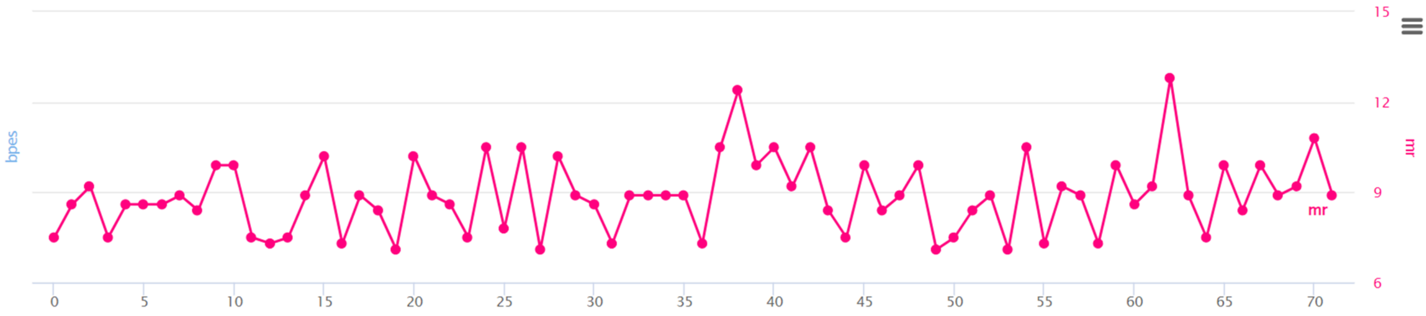

For the amino acid sequence entered by the user, the AIVE system provides a total of 6 evaluation charts.
① AIVE predicts protein structures due to mutations in each gene for coronavirus lineages or sub-lineages. From the predicted result, it carries out grouping of amino acids (components of 3D protein structure) utilizing K-means clustering to report SCPS results.
② through repeated pattern analysis of polar amino acids in amino acid sequences, AIVE reports PCS results.
③ AIVE figures out amino acid properties to measure BPES through measurement of changes in biochemical properties of amino acid.
④ AIVE calculates MR through rate of change for nucleotide frequencies due to mutations.

Result report page – APESS & distribution

⑤ APESS

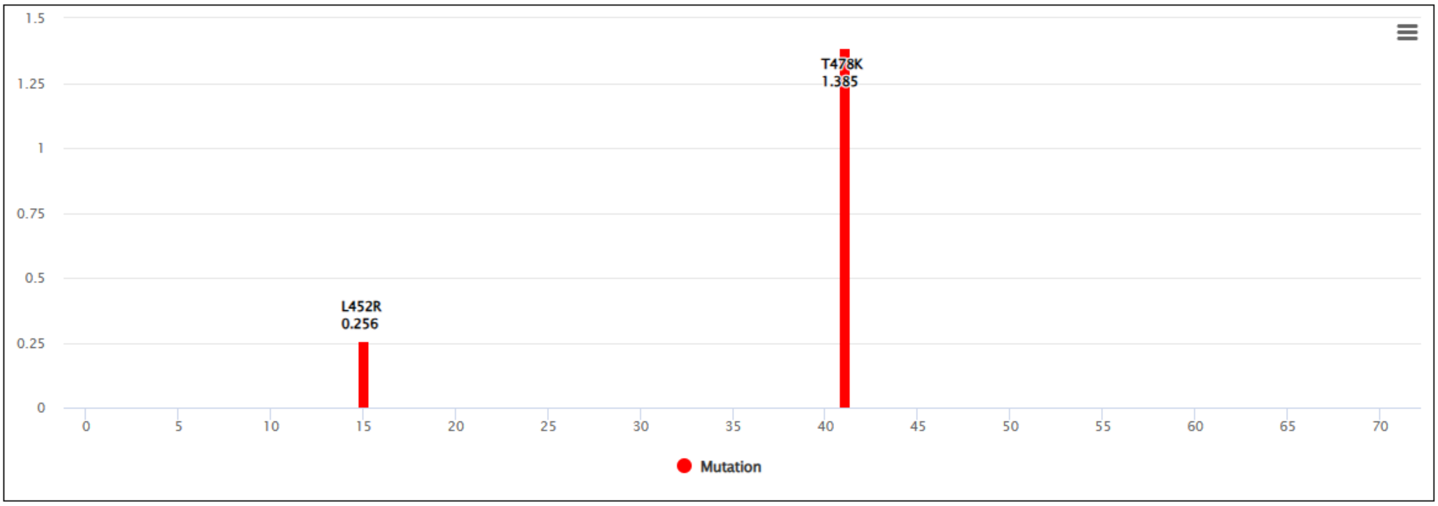

⑥ APESS distribution graph

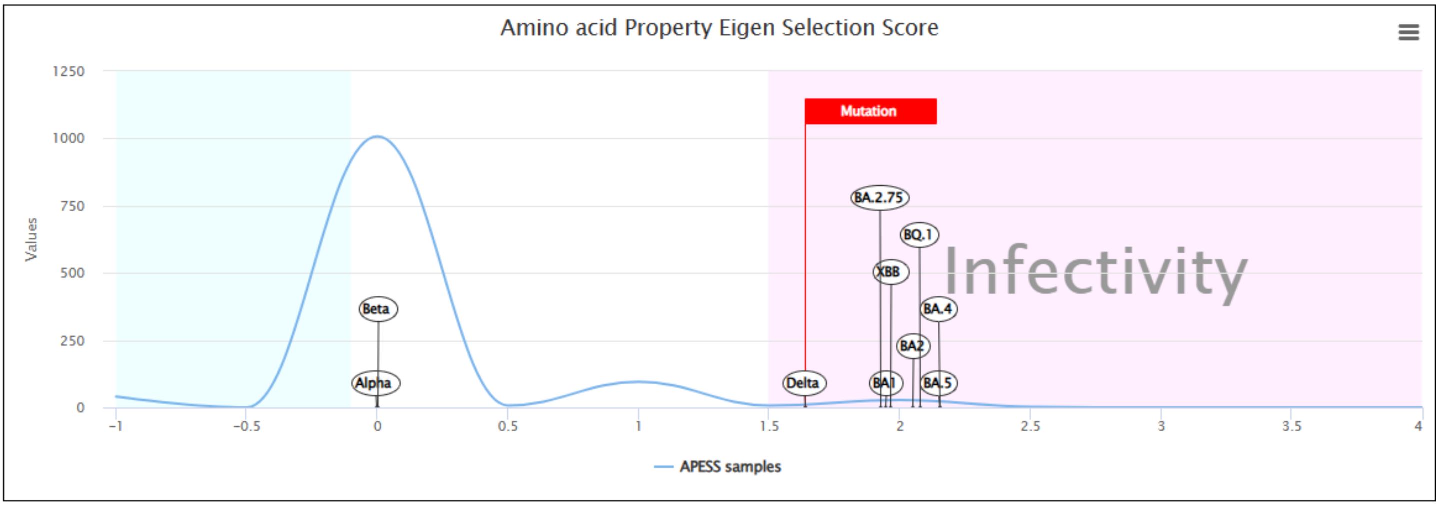

⑤ APESS, the result of comprehensive mathematical model (SCP*PCS*MR*BPES) of measured analysis results is provided.
⑥ the APESS distribution graph provides risk and spread results of the amino acid sequence entered by the user by comparing to VOCs' APESS evaluation metric.

Result report page - Polarity

⑤ Virus amino acid info

- Visualizes and tabulates changes in repeated polarity structure sequence for wild-type sequences and mutated type sequences used in ①.

Sequence without mutations (Reference)

N	S	N	N	L	D	S	K	V	G	G	N	Y	N	Y	L	Y	R	L	F	R	K	S	N
P	P	P	P	N	A	P	B	N	N	N	P	P	P	P	N	P	B	N	N	B	B	P	P
P	P	P	P	H	O	P	E	H	S	S	P	H	P	H	H	H	E	H	H	E	E	P	P

L	K	P	F	E	R	D	I	S	T	[illegible]	I	Y	Q	A	G	S	T	P	C	N	G	V	E
N	B	N	N	A	B	A	N	[illegible]	[illegible]	[illegible]	N	P	P	N	N	P	P	N	P	P	N	N	A
H	E	S	H	O	E	O	[illegible]	[illegible]	[illegible]	O	H	H	P	[illegible]	[illegible]	P	P	S	S	P	S	H	O

① ② ③

G	F	N	C	Y	F	P	L	Q	S	Y	G	F	Q	P	T	N	G	V	G	Y	Q	P	Y
N	N	P	P	P	N	N	N	P	P	P	N	N	P	N	P	P	N	N	N	P	P	N	P
S	H	P	S	H	H	S	H	P	P	H	S	H	P	S	P	P	S	H	S	H	P	S	H

Sequence with mutations (Mutation)

④

N	S	N	N	L	D	S	K	V	G	G	N	Y	N	Y	R	Y	R	L	F	R	K	S	N
P	P	P	P	N	A	P	B	N	N	N	P	P	P	P	B	P	B	N	N	B	B	P	P
P	P	P	P	H	O	P	E	H	S	S	P	H	P	H	E	H	E	H	H	E	E	P	P

L	K	P	F	E	R	D	I	S	T	E	I	Y	Q	A	G	S	K	P	C	N	G	V	E
N	B	N	N	A	B	A	N	P	P	A	N	P	P	N	N	P	B	N	P	P	N	N	A
H	E	S	H	O	E	O	H	P	P	O	H	H	P	H	S	P	E	S	S	P	S	H	O

G	F	N	C	Y	F	P	L	Q	S	Y	G	F	Q	P	T	N	G	V	G	Y	Q	P	Y
N	N	P	P	P	N	N	N	P	P	P	N	N	P	N	P	P	N	N	N	P	P	N	P
S	H	P	S	H	H	S	H	P	P	H	S	H	P	S	P	P	S	H	S	H	P	S	H

Polarity features

Polarity structure	Count	
	Reference	Mutation
ETC	41	42

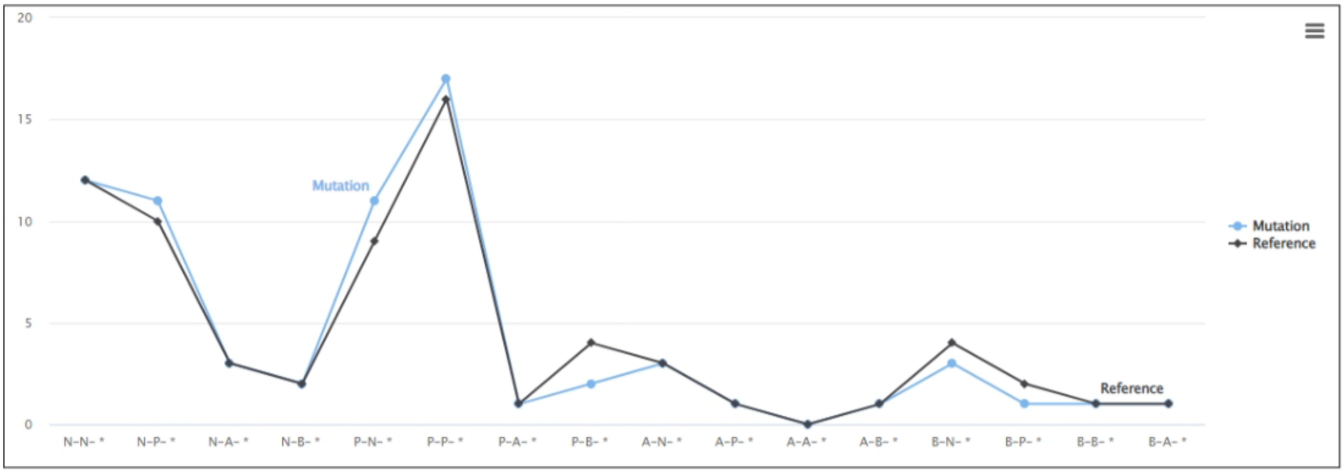

Amino acid polarity affects protein structure and stability. As a result, the amino acid polarity due to mutation of the amino acid sequence input by the user can be observed. We found repeated polarity patterns in the coronavirus and observed changes in the properties of amino acid sequence polarity due to mutation. Therefore, we provide visualization and table view of polarity pattern changes to the user.

① amino acid sequence
② 4 polarity characteristics
③ 5 amino acid properties
④ Mutated positions are indicated in red.

Result report page – All viruses

Prediction Result

XBB

① 3D Structure Prediction ⓘ

- The PAE is a value that estimates the difference between the relative locations of two residues of the model and the real model. The value has a negative correlation with the accuracy of the pairwise position of two residues. As the PAE value decreases, the accuracy increases and vice versa.
- The pLDDT is a value that estimates the reliability of the model. The value indicates the likelihood of folding of the protein structure at that location, with higher values indicating a greater likelihood of folding.

1 2 3 4 5 ⓘ Download all file

Protein structure (Mutation) Compare

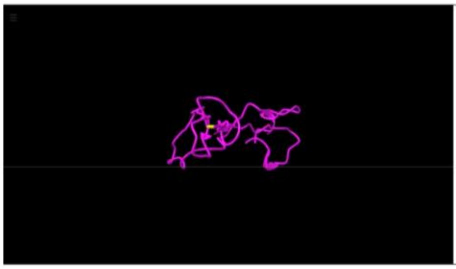

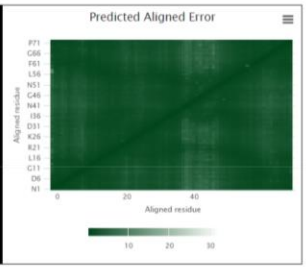

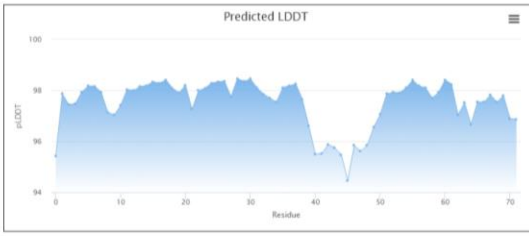

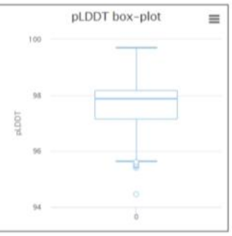

② Virus amino acid info

- Visualizes and tabulates changes in repeated polarity structure sequence for wild-type sequences and mutated type sequences used in ①.

Sequence without mutations (Reference)

N	S	N	K	L	D	S	K	P	S	G	N	Y	N	Y	L	Y	R	L	F	R	K	S	K	L	K	P	F	E	R	D	I	S	T	E	I	Y	Q	A	G	N	K	P	C	N	G	V	A
P	P	P	B	N	A	P	B	N	P	N	P	P	P	P	N	P	B	N	N	B	B	P	B	N	B	N	N	A	B	A	N	P	P	A	N	P	P	N	N	P	B	N	P	P	N	N	N
P	P	P	E	H	O	P	E	S	P	S	P	H	P	H	H	H	E	H	H	E	E	P	E	H	E	S	H	O	E	O	H	P	P	O	H	H	P	H	S	P	E	S	S	P	S	H	H

G	S	N	C	Y	S	P	L	Q	S	Y	G	F	R	P	T	Y	G	V	G	H	Q	P	Y
N	P	P	P	P	P	N	N	P	P	P	N	N	B	N	P	P	N	N	N	B	P	N	P
S	P	P	S	H	P	S	H	P	P	H	S	H	E	S	P	H	S	H	S	E	P	S	H

Sequence with mutations (Mutation)

N	S	N	K	L	D	S	K	P	S	G	N	Y	N	Y	L	Y	R	L	F	R	K	S	K	L	K	P	F	E	R	D	I	S	T	E	I	Y	Q	A	G	N	K	P	C	N	G	V	A
P	P	P	B	N	A	P	B	N	P	N	P	P	P	P	N	P	B	N	N	B	B	P	B	N	B	N	N	A	B	A	N	P	P	A	N	P	P	N	N	P	B	N	P	P	N	N	N
P	P	P	E	H	O	P	E	S	P	S	P	H	P	H	H	H	E	H	H	E	E	P	E	H	E	S	H	O	E	O	H	P	P	O	H	H	P	H	S	P	E	S	S	P	S	H	H

G	S	N	C	Y	S	P	L	Q	S	Y	G	F	R	P	T	Y	G	V	G	H	Q	P	Y
N	P	P	P	P	P	N	N	P	P	P	N	N	B	N	P	P	N	N	N	B	P	N	P
S	P	P	S	H	P	S	H	P	P	H	S	H	E	S	P	H	S	H	S	E	P	S	H

Polarity feature			Amino acid
Polarity features	Non-Polar	N	Ala (A), Val (V), Leu (L), Gly (G), Ile (I), Met (M), Trp (W), Phe (F), Pro (P)
	Polar	P	Ser (S), Cys (C), Asn (N), Gln (Q), Thr (T), Tyr (Y)
	Acidic	A	Asp (D), Glu (E)
	Basic	B	Lys (K), Arg (R), His (H)
Five amino acid properties	Amino acids with electrically charged side chain_negative	1	Lys (K), Arg (R), His (H)
	Amino acids with electriccally charged side chain_positive	2	Asp (D), Glu (E)
	Amino acids with Polar uncharged side chain	3	Ser (S), Asn (N), Gln (Q), Thr (T)
	Special cases	4	Cys (C), Gly (G), Pro (P)
	Amino acids with hydrophobic side chain	5	Ala (A), Val (V), Leu (L), Ile (I), Met (M), Trp (W), Phe (F)

Polarity features

Polarity structure	Count	
	Reference	Mutation
ETC	45	45

Result report page –Compare

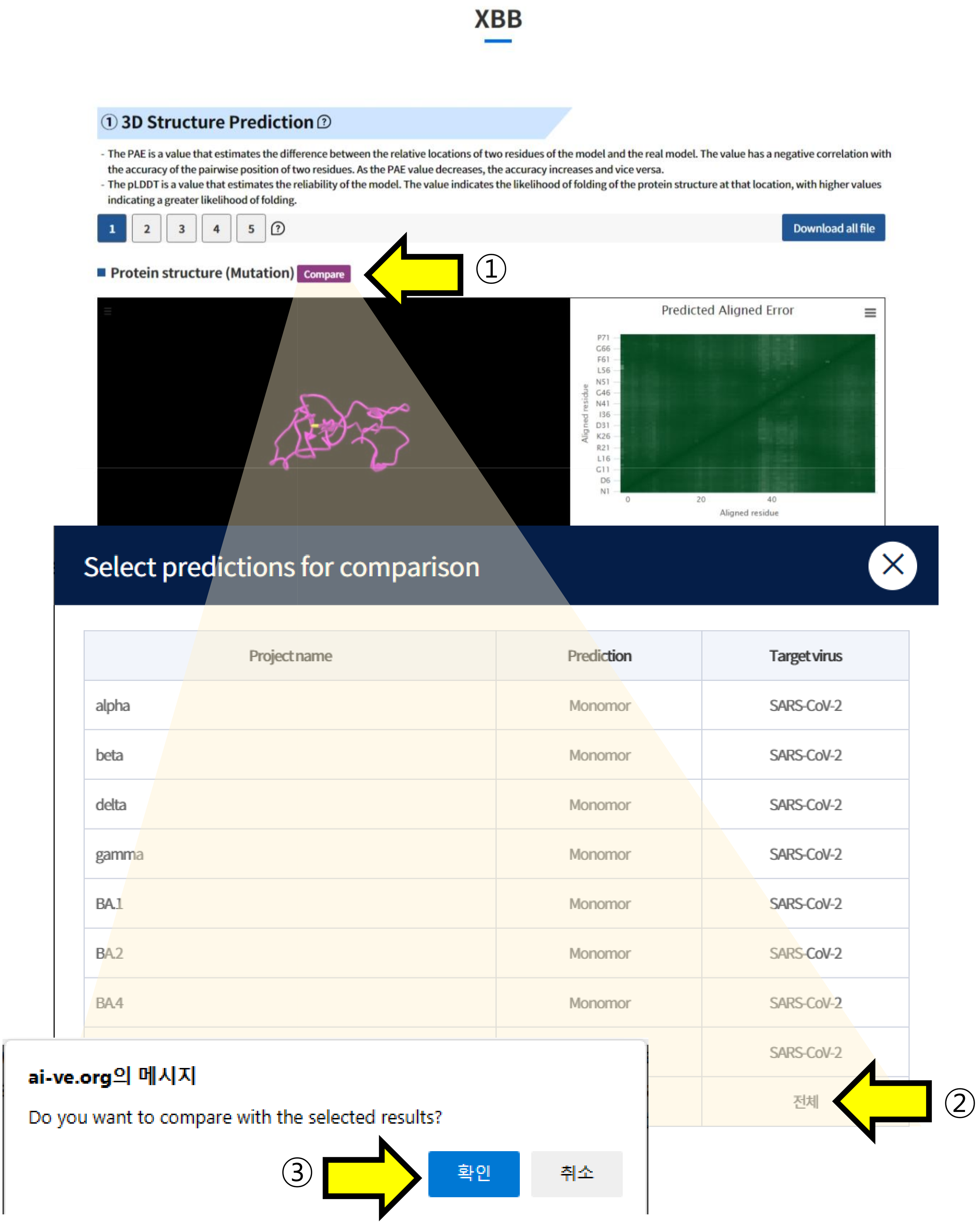

Structures predicted by “All viruses” can be compared with other structures using the compare feature.
① Click the "Compare" button to load the list of other tasks submitted by the user.
② Select the task you want to compare and click on it.
③ Click the "Confirm" button to navigate to the comparison page.

Result report page –Compare

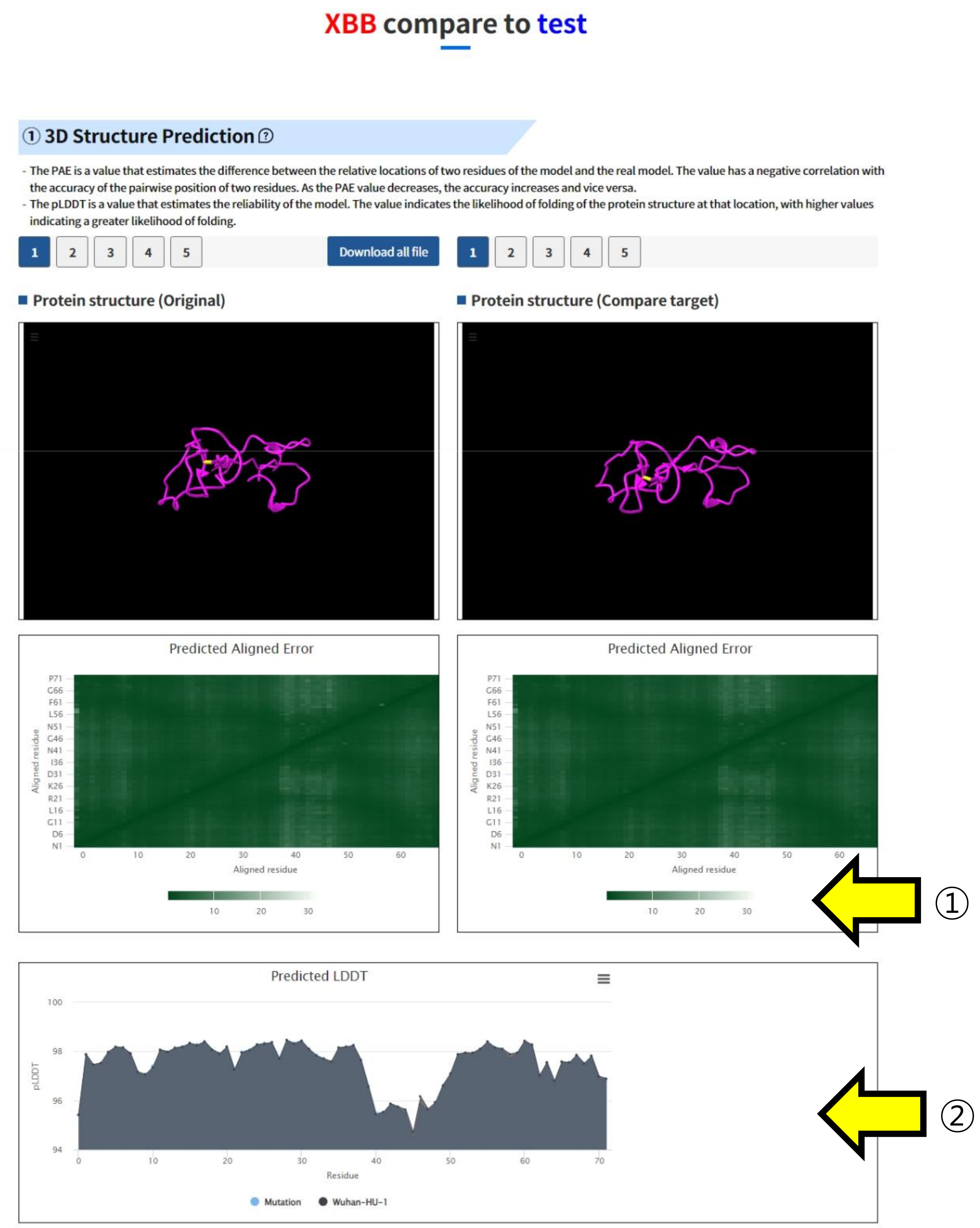

Through the "Compare" feature, you can compare two predicted structures in the list:
① You can visually inspect the PAE (Predicted Alignment Error) of the two structures using a plot.
② You can compare the pLDDT values of the two structures.

Result report page –Compare

③

② Virus amino acid info

- Visualizes and tabulates changes in repeated polarity structure sequence for wild-type sequences and mutated type sequences used in ①.

■ Sequence without mutations (Original)

N	S	N	K	L	D	S	K	P	S	G	N	Y	N	Y	L	Y	R	L	F	R	K	S	K	L	K	P	F	E	R	D	I	S	T	E	I	Y	Q	A	G	N	K	P	C	N	G	V	A
P	P	P	B	N	A	P	B	N	P	N	P	P	P	P	N	P	B	N	N	B	B	P	B	N	B	N	N	A	B	A	N	P	P	A	N	P	P	N	N	P	B	N	P	P	N	N	N
P	P	P	E	H	O	P	E	S	P	S	P	H	P	H	H	H	E	H	H	E	E	P	E	H	E	S	H	O	E	O	H	P	P	O	H	H	P	H	S	P	E	S	S	P	S	H	H

↓

G	S	N	C	Y	S	P	L	R	S	Y	G	F	R	P	T	Y	G	V	G	H	Q	P	Y
N	P	P	P	P	P	N	N	B	P	P	N	N	B	N	P	P	N	N	N	B	P	N	P
S	P	P	S	H	P	S	H	E	P	H	S	H	E	S	P	H	S	H	S	E	P	S	H

■ Sequence with mutations (Compare target)

N	S	N	K	L	D	S	K	P	S	G	N	Y	N	Y	L	Y	R	L	F	R	K	S	K	L	K	P	F	E	R	D	I	S	T	E	I	Y	Q	A	G	N	K	P	C	N	G	V	A
P	P	P	B	N	A	P	B	N	P	N	P	P	P	P	N	P	B	N	N	B	B	P	B	N	B	N	N	A	B	A	N	P	P	A	N	P	P	N	N	P	B	N	P	P	N	N	N
P	P	P	E	H	O	P	E	S	P	S	P	H	P	H	H	H	E	H	H	E	E	P	E	H	E	S	H	O	E	O	H	P	P	O	H	H	P	H	S	P	E	S	S	P	S	H	H

↓

G	S	N	C	Y	S	P	L	Q	S	Y	G	F	R	P	T	Y	G	V	G	H	Q	P	Y
N	P	P	P	P	P	N	N	P	P	P	N	N	B	N	P	P	N	N	N	B	P	N	P
S	P	P	S	H	P	S	H	P	P	H	S	H	E	S	P	H	S	H	S	E	P	S	H

Polarity feature			Amino acid
Polarity features	Non-Polar	N	Ala (A), Val (V), Leu (L), Gly (G), Ile (I), Met (M), Trp (W), Phe (F), Pro (P)
	Polar	P	Ser (S), Cys (C), Asn (N) , Gln (Q), Thr (T), Tyr (Y)
	Acidic	A	Asp (D), Glu (E)
	Basic	B	Lys (K), Arg (R), His (H)
Five amino acid properties	Amino acids with electrically charged side chain_negative	1	Lys (K), Arg (R), His (H)
	Amino acids with electriccally charged side chain_positive	2	Asp (D), Glu (E)
	Amino acids with Polar uncharged side chain	3	Ser (S), Asn (N) , Gln (Q), Thr (T)
	Special cases	4	Cys (C), Gly (G), Pro (P)
	Amino acids with hydrophobic side chain	5	Ala (A), Val (V), Leu (L), Ile (I), Met (M), Trp (W), Phe (F)

■ Polarity features

Polarity structure	Count	
	Original	Compare target
ETC	46	45

③ The sequence and polar structures of the two structures can be compared and analyzed.
